# Risk factors for frailty in older adults

**DOI:** 10.1097/MD.0000000000030169

**Published:** 2022-08-26

**Authors:** Xinrui Wang, Jiji Hu, Diping Wu

**Affiliations:** a Department of Nursery, Gulin people’s Hospital, Sichuan, China.

**Keywords:** ageing adults, frailty, meta-analysis, risk factors

## Abstract

To clarify the risk factors for frailty to help doctors prevent diseases that cause weakness, through early interventions.

**Methods:**

We searched the PubMed, EMBASE, and Cochrane Library databases to identify all relevant studies using the items “frailty,” “weak,” “risk factors,” and “predictive factors” and compared their results. The aging population (≥65 years old) was divided into 2 groups, a “frailty group” and a “robust control group,” and then the characteristics, lifestyles, and comorbidities were compared.

**Results:**

We compared the influence of baseline and concomitant diseases on frailty in the elderly respectively, and the analysis of the influence of baseline on frailty found that increasing age, lower weight, female sex, living alone, low levels of exercise, polypharmacy, higher education level, smoking, drinking, malnutrition, and lower vitamin D levels were associated with aging individuals being more likely to experience frailty. The data about concomitant diseases had shown that diabetes, hearing dysfunction, cognitive impairment, poor sleep, a history of falls, pain, and depression can increase the risk of frailty among the elderly population.

**Conclusion:**

Characteristics, comorbidities, and lifestyle factors can impact the occurrence of frailty, and relevant influencing factors should be considered.

## 1. Introduction

The ageing of the population worldwide is an important public health issue. The increasing frailty of elderly individuals that has accompanied this trend has become a major problem in the field of population ageing.^[1]^ This is mostly because of the association of frailty with an increased risk of adverse health outcomes.^[2,3]^ Physical frailty is a biological syndrome that reflects a diminished reserve to buffer against stressors due to deteriorating physiological systems that results in vulnerability to adverse health consequences and inducing cumulative decline in multiple physiologic systems and that has been associated with an increased risk of falls, disability, institutionalization, hospitalization, systemic diseases, and all-cause mortality.^[1,4]^ In general, the definition of frailty is a condition of increased vulnerability to stressor events as a consequence of the cumulative decline in many physiological systems,^[5]^ and the factors that can predict the occurrence of frailty deserve deep exploration.^[4]^ Among numerous frailty-assessment tools, the most popular models are the physical frailty phenotype operationalized in 2001 by Fried et al^[6]^ based on 5 frailty criteria, until Rockwood cumulative deficit model proposed, that is a frailty index based on a comprehensive geriatric assessment of individual deficits.^[7]^ The definition used in this paper was based on the updated tool created by Rockwood and Mitnitski^[7]^ in 2011. Several recent studies have identified severity of frailty across various conditions, including old age, female sex, and reduced concentration and motivation.^[4,5,8–41]^ However, high-quality systematic reviews are still in demand. Overall, we conducted this project to study the risk factors for frailty.

## 2. Methods

### 2.1. Ethical review

Ethical approval was not necessary, because this is a review.

### 2.2. Search strategy and data extraction

This research of this systematic review was performed according to PRISMA checklist. We searched for articles published in recently decade (till January 2022), since we performed the definition of frailty proposed by Rockwood et al in 2011.^[7]^ And the initial search process was designed to find all reports involving with the search terms: (“risk factors” OR “predictive factors”) AND (“Frailty” OR “weak”). These searches were conducted in the following databases: PubMed, Web of Science, MEDLINE, EMBASE, and Google Scholar. A manual search was also performed to compensate for the deficiency of computer searches. The definition used in this paper was based on the updated tool created by Rockwood and Mitnitski^[7]^ in 2011. Two authors independently screened all citations and abstracts identified by the search strategy to select potentially eligible studies. Data were independently extracted by 2 authors using a predesigned data extraction Excel file. The inclusion criteria are: paper should with 2 groups: aging people (≥65 years old) with frailty and without frailty (definition of frailty according to Rockwood and Mitnitski^[7]^); and the study contained in this project should present the data about baseline Characteristics and lifestyles of people, Comorbidities condition of people; all the definitions found for frailty were homogeneous in the selected articles. The exclusion criteria are: studies with redundant publications, reviews, unrelated topic, different frailty criteria, and different group settings.

### 2.3. Comparisons and quality assessment

Finally, there are 36 studies^[4,5,8–41]^ with 58,028 people included in this systematic-review. Data extraction included the baseline characteristics of aging adults, their lifestyle habits, their accompanying diseases, and whether they had experienced falls. And we evaluated 22 meta-analyses and compared 2 groups (frailty and healthy control groups) in each of the meta-analyses. The 22 meta-analyses included several risk factors including the following: age, body mass index (BMI), sex (female), living alone, low levels of exercise, polypharmacy (which was defined as not less than 5 kinds of oral prescription drugs^[16]^), the recorded number of drugs, education, smoking, drinking, malnutrition, vitamin D levels, and comorbidity (which was defined when the total number of recorded diseases of participant was greater than or equal to 2^[12]^). In addition, ten comorbid diseases were compared between people with frailty and those without frailty: stroke, cardiac disease, diabetes, vision dysfunction, hearing dysfunction, cognitive impairment, poor sleep, fall history, pain, and depression.

The quality of the included studies was assessed by 2 authors, according to the Cochrane Collaboration Reviewer’s Handbook and the Quality of Reporting of Meta-analysis guidelines.^[42,43]^

### 2.4. Data analysis

All of the final information was subsequently entered into Review Manager (RevMan) 5.1.4, which is Cochrane software for preparing and maintaining Cochrane reviews. It can perform meta-analysis of the data entered, and present results graphically. Continuous outcomes are presented as weighted mean differences with 95% confidence intervals (CIs). Dichotomous data are presented as relative risk (RR) with 95% CIs. The analysis of the meta-analyses was performed using the fixed-effects or random-effects method. The fixed-effects method was used to combine the results when no significant heterogeneity was present. The random-effects method was applied when heterogeneity was present. Statistical heterogeneity among the trials was evaluated using the *I*^2^ test (the iterative noncentral chi-square distribution method), with significance set at *P* < .05.

## 3. Results

### 3.1. Description of the included studies

**Figure 1.**
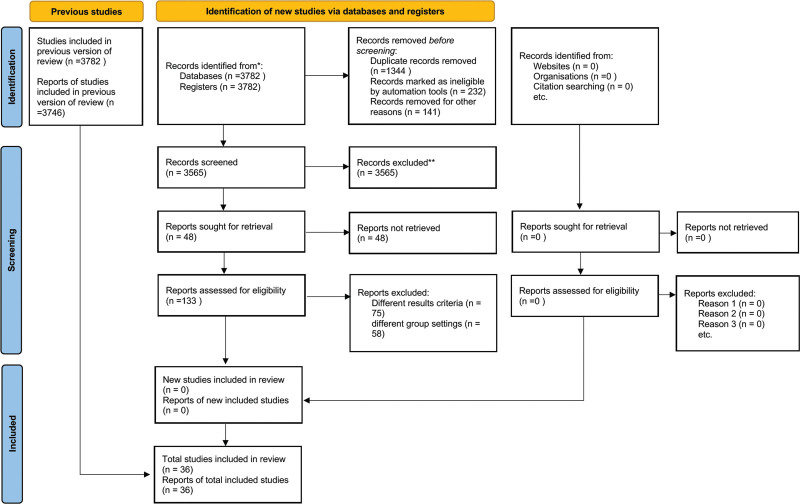
Flowchart of literature review and reasons for exclusion.

A total of 3778 reports were initially identified from the database and manual search. At first step, 3565 reports were excluded from the study for the following reason: redundant publications, reviews, unrelated topic. After referring to the full text, 95 articles with different frailty criteria, and 82 articles with different group settings were excluded. We eventually included 36 papers for this research.^[4,5,8–41]^ The conditions in these studies and the clinical details of the patients are presented in Table 1. The search flow diagram is presented in Figure 1.

**Table 1:** Summary of included papers.

Author	Year	Included number	Research type
Arts	2021	378	Retrospect study
Ayesta	2021	98	Retrospect study
Brutto	2020	248	Retrospect study
Chen	2021	85	Retrospect study
Fan	2020	454	Retrospect study
Gilmore	2021	319	Retrospect study
Gomes	2018	804	Retrospect study
Henchoz	2017	634	Retrospect study
Hong	2019	299	Retrospect study
Ikeda	2019	25,549	Retrospect study
Jang	2021	2340	Retrospect study
Jiao	2020	9996	Retrospect study
Jung	2020	2907	Retrospect study
Kim	2021	252	Retrospect study
Kume	2021	150	Retrospect study
Lee	2014	1104	Retrospect study
Lee	2021	3040	Retrospect study
Liang	2021	179	Retrospect study
Liu	2021	7442	Retrospect study
McKechnie	2021	981	Retrospect study
Moradell	2021	33	Retrospect study
Okamura	2021	1565	Retrospect study
Oyon	2021	338	Retrospect study
Ozsoy	2021	166	Retrospect study
Poli	2017	361	Retrospect study
Rizka	2021	214	Retrospect study
Setiati	2021	888	Retrospect study
Sharma	2021	243	Retrospect study
Tamayo	2017	284	Retrospect study
Tang	2021	345	Retrospect study
Valdiviesso	2021	58	Retrospect study
Wang	2021	2363	Retrospect study
Xu	2021	642	Retrospect study
Yang	2018	147	Retrospect study
Yuan	2021	4894	Retrospect study
Zhao	2021	452	Retrospect study

### 3.2. Characteristics and lifestyles of people with/without frailty

**Figure 2.**
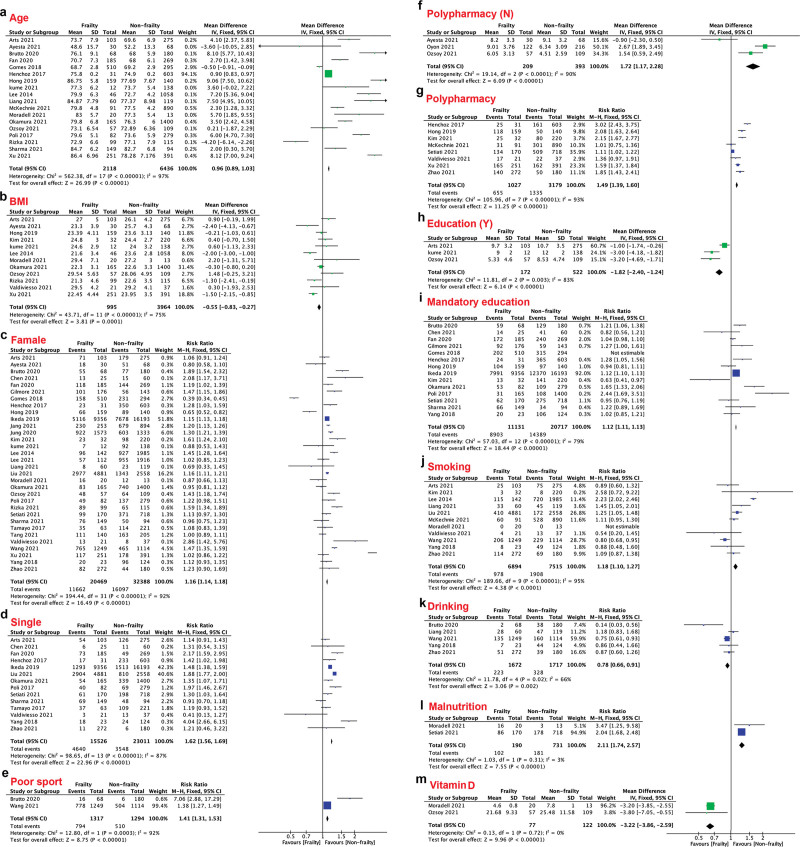
The forest plots of the impact of characteristics on frailty.

First, we compared higher age, higher BMI, sex (female), living alone, low levels of exercise, polypharmacy, low level education, smoking, drinking, malnutrition and lower vitamin D levels among ageing people (Fig. 2A–M). Older people (RR: 0.96, 95% CI: 0.89–1.03, *P* < .001; Fig. 2A), lower BMI (RR: −0.55, 95% CI: −0.83– to 0.27, *P* < .001; Fig. 2B), female sex (RR: 1.16, 95% CI: 1.14–1.18, *P* < .001; Fig. 2C), single (RR: 1.62, 95% CI: 1.56–1.69, *P* < .001; Fig. 2D), low levels of exercise (RR: 1.41, 95% CI: 1.31–1.53, *P* < .001; Fig. 2E), polypharmacy (RR: 1.72, 95% CI: 1.17–2.28, *P* < .001; RR: 1.49, 95% CI: 1.39–1.60, *P* < .001; Fig. 2F, G), smoking (RR: 1.18, 95% CI: 1.10–1.27, *P* < .001; Fig. 2J), drinking (RR: 0.78, 95% CI: 0.66–0.91, *P* = .002; Fig. 2K), malnutrition (RR: 2.11, 95% CI: 1.74–2.57, *P* < .001; Fig. 2L), and lower vitamin D levels (RR: −3.22, 95% CI: −3.86 to 2.59, *P* < .001; Fig. 2M) were associated with frailty. The association between education level and frailty remains inconsistent: a longer education duration decreased the risk of frailty (RR: −1.82, 95% CI: −2.40 to 1.24, *P* < .001; Fig. 2H), while the population that completed the mandatory education was more likely to become frail (RR: 1.12, 95% CI: 1.11–1.13, *P* < .001; Fig. 2I).

### 3.3. Comorbidities of people with/out frailty

**Figure 3.**
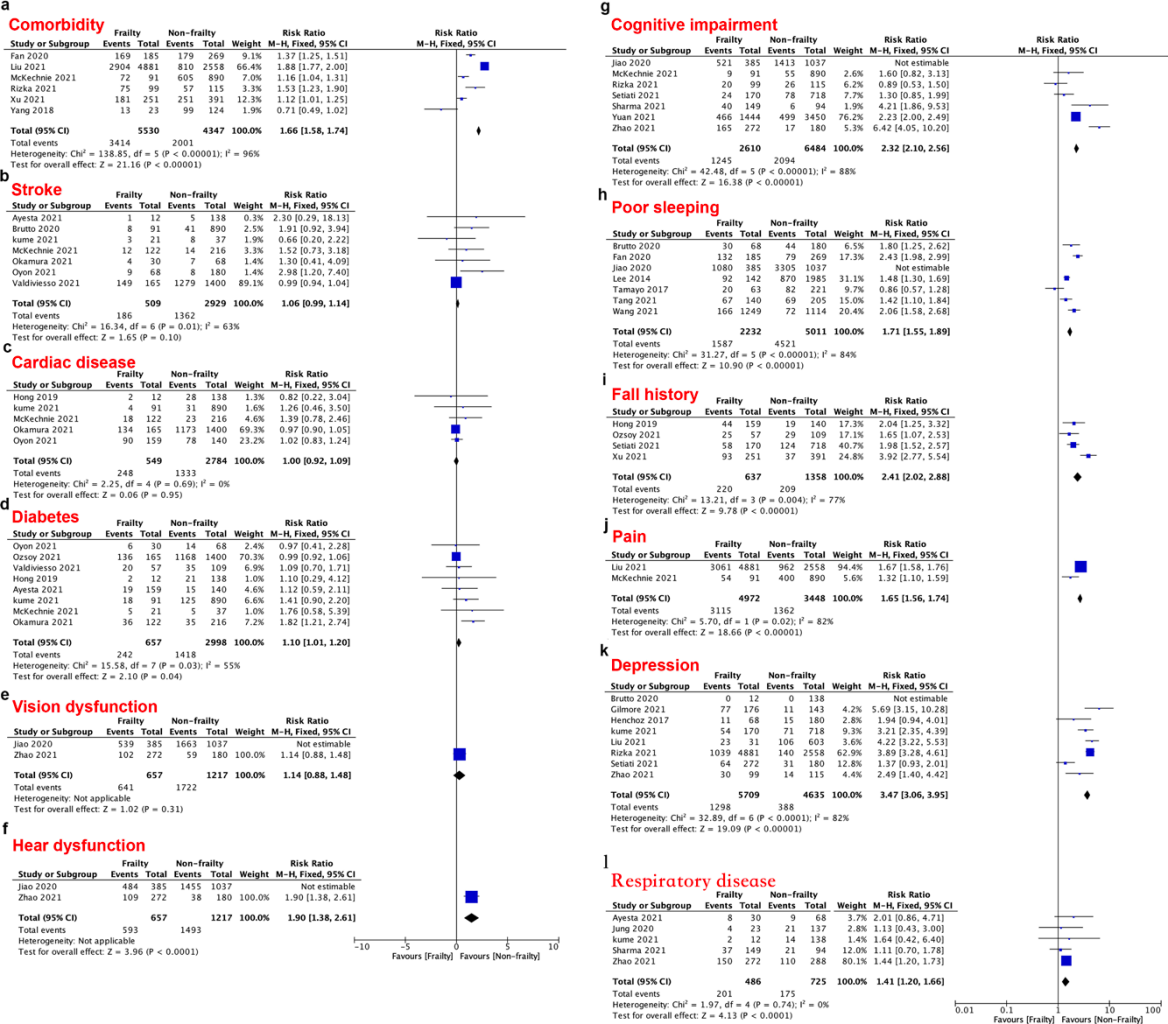
The forest plots of the impact of lifestyle and comorbidities on frailty.

The comorbidities and comorbid diseases were compared (Fig. 3A–K). Even though comorbidities can actually impact the risk of frailty (RR: 1.66, 95% CI: 1.58–1.74, *P* < .001; Fig. 3A), not all of the conditions mentioned above affected frailty. Stroke (RR: 1.06, 95% CI: 0.99–1.14, *P* = .10; Fig. 3B), cardiac disease (RR: 1.00, 95% CI: 0.92–1.09, *P* = .95; Fig. 3C) and vision dysfunction (RR: 1.14, 95% CI: 0.88–1.48, *P* = .31; Fig. 3E) were not significantly different between the 2 groups. However, diabetes (RR: 1.10, 95% CI: 1.01–1.20, *P* = .04; Fig. 3D), hearing dysfunction (RR: 1.90, 95% CI: 1.38–2.61, *P* < .001; Fig. 3F), cognitive impairment (RR: 2.32, 95% CI: 2.10–2.56, *P* < .001; Fig. 3G), poor sleep (RR: 1.71, 95% CI: 1.55–1.89, *P* < .001; Fig. 3H), fall history (RR: 2.41, 95% CI: 2.02–2.88, *P* < .001; Fig. 3I), pain (RR: 1.65, 95% CI: 1.56–1.74, *P* < .001; Fig. 3J) and depression (RR: 3.47, 95% CI: 3.06–3.95, *P* < .001; Fig. 3K) can increase the risk of frailty among ageing people, respiratory disease (RR: 1.41, 95% CI: 1.20–1.66, *P* < .001; Fig. 3L).

## 4. Discussion

Frailty is a very large challenge that occurs with a rapidly expanding older population. The overall prevalence of frailty in this community-dwelling population was 6.9%; frailty increased with age and was greater in female than in male. Four-year incidence under the frailty circumstance was 7.2%.^[18]^ It was characterized by increased vulnerability to stressors following an acceleration in the gradual decrease in physiological function, and it can impose a heavy burden on frail older adults, care professionals, and healthcare systems.^[6,15,44]^ Therefore, several clinical guidelines for frailty management have suggested that modifiable risk factors for frailty, such as unhealthy diet and insufficient physical activity, should be addressed earlier.^[36]^ The aim of this study was to evaluate the associations among frailty and basic characteristics, lifestyle factors, and comorbidities, with a specific focus on preventing the serious consequences of frailty. The collection of studies included in this systematic review contained a large and representative sample of community-dwelling older people.

According to our results, frailty was associated with older age, lower BMI, female sex, living alone, low levels of exercise, polypharmacy, smoking status, drinking status, low vitamin D levels, and malnutrition. These factors have been reported in several papers. The association with higher education level was controversial, as a longer period of education can decrease the likelihood of frailty (*P* < .001), while the population that completed the mandatory education was more likely to become frailty. However, the definition of mandatory is found to be different among several reports. Therefore, we assume that education time may decline frailty rate. Education is a social factor, and studies have only recently demonstrated the influence of social factors in the onset of frail conditions rather than limiting the approach to frailty to within a biological framework.^[45,46]^ In particular, among the different lifestyle factors, we still know very little about cultural/social influences. Thus, identifying this factor and its potential role in the pathophysiology of frailty becomes of great importance to establish multidimensional models of prevention and treatment. This may help us understand the relationship between frailty and education level. In summary, this issue deserves further exploration in the future.

In addition to the physiological vulnerability caused by ageing, older adults who live alone are often vulnerable in terms of physical and psychosocial aspects.^[47]^ Living alone is an important question that people face, and it is increasing year after year. Compared to those who live with their spouse or children, older adults who live alone have been associated with a lower economic status, a higher prevalence of chronic diseases, multimorbidity, depressive symptoms, and a higher percentage of safety incidents (falls and abuse).^[47]^ For those reasons, older adults living alone are more vulnerable to physical, mental, and social impacts on health, which highlights the need for societal attention and support to help them maintain multilateral aspects of health and function as well as their independence.^[47]^

There are several previously published meta-analysis reports which all discussed the risk factors of frailty, but they focused only on the risk factors for frailty in specific populations. For instance, the research of Burton et al^[48]^ only focused on the risk factors of frailty in acute stroke, and they only include 14 papers. The paper of Gallo et al^[49]^ only focused on the risk factors of frailty in plastic surgery, and the study of Liu et al^[50]^ only focused on the preoperative and postoperative delirium. While the report of Hajek et al^[51]^ only contained the personality factors, we also include the effects of concomitant diseases on weakness. Above all, we have focused on risk factors for frailty in all elderly populations, which may help to better monitor the health of elderly patients; this is our advantage. However, some limitations must also be mentioned. First, the sociological factors affecting Frailty need to be further explored; second, we did not further compare the difference in frailty between community-dwelling elderly individuals and elderly individuals in the hospital. Finally, more research is needed regarding interventions for this form of frailty.

## 5. Conclusion

The final results demonstrated the following: among the ageing population, older age, low BMI, female sex, living alone, low levels of exercise, polypharmacy, education, smoking, drinking, malnutrition, and low vitamin D levels had significant relationships with frailty; elderly adults with diabetes, hearing dysfunction, cognitive impairment, poor sleep, a history of falls, pain, and depression were at a higher risk of frailty than those without those comorbidities.

## Author contributions

**Data curation:** Diping Wu.

**Investigation:** Diping Wu, Xinrui Wang.

**Methodology:** Xinrui Wang.

**Supervision:** Diping Wu, Jiji Hu.

**Writing – original draft:** Jiji Hu.
